# Evaluation of imaging setups for quantitative phase contrast nanoCT of mineralized biomaterials

**DOI:** 10.1107/S1600577522003137

**Published:** 2022-04-25

**Authors:** Jussi-Petteri Suuronen, Bernhard Hesse, Max Langer, Marc Bohner, Julie Villanova

**Affiliations:** a Xploraytion, Bismarckstrasse 10-12, 10625 Berlin, Germany; b ESRF – The European Synchrotron, 71 Avenue des Martyrs, 38043 Grenoble, France; c University of Lyon, INSA-Lyon, Université Claude Bernard Lyon 1, UJM-Saint Etienne, CNRS, Inserm, CREATIS UMR 5220, U1206, 69621 Lyon, France; d University of Grenoble Alpes, CNRS, UMR 5525, VetAgro Sup, Grenoble INP, TIMC, 38000 Grenoble, France; e RMS Foundation, Bischmattstrasse 12, 2544 Bettlach, Switzerland

**Keywords:** 3D X-ray nano-tomography, resolution, image quality, bone, scaffold, synchrotron radiation imaging

## Abstract

X-ray nano-tomography with phase contrast (nanoCT) using synchrotron radiation is a powerful tool to non-destructively investigate 3D material properties at the nanoscale. In large bone lesions, such as severe bone fractures, bone cancer or other diseases, bone grafts substituting the lost bone might be necessary. Such grafts can be of biological origin or be composed of a synthetic bone substitute. The long-term functioning of artificial bone substitutes depends on many factors. Synchrotron nanoCT imaging has great potential to contribute to further the understanding of integration of implants into bone tissue by imaging the spatial interaction between bone tissue and implant, and by accessing the interface between implant material and bone tissue. With this aim, a methodology for evaluating the image quality is presented for in-line phase contrast nanoCT images of bone scaffold samples. A PMMA-embedded tricalcium phosphate scaffold was used with both a closed and an open porosity structure and bone ingrowths as a representative system of three known materials. Parameters such as spatial resolution and signal-to-noise ratio were extracted and used to explore and quantitatively compare the effects of implementation choices in the imaging setup, such as camera technology and imaging energy, on the resulting image quality. Increasing the X-ray energy from 17.5 keV to 29.6 keV leads to a notable improvement in image quality regardless of the camera technology used, with the two tested camera setups performing at a comparable level when the recorded intensity was kept constant.

## Abstract 2

This work evaluates the impact of experimental conditions on image quality when performing synchrotron X-ray in-line phase contrast nano-tomography on mineralized biomaterials. For this purpose, a computational procedure to investigate signal-to-noise ratio and spatial resolution is proposed.

## 1. Introduction

X-ray nano-tomography (nanoCT) with phase contrast using synchrotron radiation is a powerful tool for the non-destructive investigation of 3D material properties at the nanoscale (Mokso *et al.*, 2007 ▸). It has enabled new insights into the mineralization process of bone and the structure and function of the lacunar-canalicular cell network (LCN) (Varga *et al.*, 2015 ▸; Wittig *et al.*, 2019 ▸). Although the LCN contributes less to overall bone porosity compared with blood vessel pores (Haversian and Volkmann canals), the surface area formed by the LCN is more than one order of magnitude larger than the surface formed by blood vessel pores (Hesse *et al.*, 2015 ▸). Mineral exchange is likely to occur at all bone interfaces including blood vessel pores and at the LCN (Ayoubi *et al.*, 2020 ▸; Bortel *et al.*, 2021 ▸; Vahidi *et al.*, 2021 ▸). The role of the osteocyte network, housed inside the LCN, is not only to contribute to mineral homeostasis through exchange of ions at their mineral interfaces but also in mechano-transduction and subsequent triggering of osteoblast/osteoclast remodeling (Cao *et al.*, 2020 ▸).

In large bone lesions, such as severe bone fractures, bone cancer or other diseases, bone grafts that substitute the lost bone can become necessary. Such grafts can be of biological origin, such as autografts, or be composed of a synthetic bone substitute. In several surgical procedures, implants can be used to stabilize the bone. The long-term functioning of artificial bone substitutes and implant materials depends on many factors. For metal implant materials, the effect of implant related particle and ion exposure in the peri-implant bone tissue is so far not fully understood (Schoon *et al.*, 2020 ▸; Nelson *et al.*, 2020 ▸). It remains an open question to what extent metal integration into bone directly alters bone tissue homeostasis induced by alterations of the LCN. In autologous bone transplantation or transplantation of degradable implant materials, the integration of newly formed bone tissue into the zones of the implant regions depends not only on ingrowth of blood vessels (Chen *et al.*, 2020 ▸) but also on access via the different bone cells into these regions (Bohner *et al.*, 2017 ▸). The investigation of the pore network structure on the cellular length scale is therefore of relevance to assess the outcome of bone grafts using new materials.

With improved hardware and software solutions, synchrotron nanoCT imaging has great potential to further the understanding of integration of metals and biomaterials into bone tissue and to characterize the interface of exogenous materials and bone tissue. However, since access to synchrotron sources is limited, an optimized compromise between image quality and scanning time, size of the field of view (FOV) and spatial resolution, and optimized contrast for different material phases is desirable.

Several different methods exist to perform 3D X-ray nano-imaging (Withers, 2007 ▸; Langer & Peyrin, 2016 ▸): ptychographic tomography (Ciani *et al.*, 2018 ▸), transmission X-ray microscopy (TXM; Andrews *et al.*, 2010 ▸) and in-line phase tomographic microscopy (Langer *et al.*, 2012 ▸). Parallel-beam projection systems are not considered here, since they cannot provide a resolution better than a few hundred nanometres (Vojtovà *et al.*, 2019 ▸; Khoury *et al.*, 2015 ▸). Ptychographic tomography can yield very high spatial resolution [∼15 nm (da Silva *et al.*, 2019 ▸)] but presents limitations such as radiation damage to the sample and long acquisition time (Ciani *et al.*, 2018 ▸; Langer & Peyrin, 2016 ▸). TXM is a set up that is increasingly common at synchrotron sources (see Table 1 ▸).

Then, even with limited, competitive access to synchrotron beamlines, the increase in TXM setups available for 3D imaging makes experiment access easier. Note that this configuration becomes more and more accessible using laboratory devices (Lu *et al.*, 2019 ▸), even if scan times remain very long. It allows a pixel size of 33 nm to be reached with an energy below 14 keV. This quite low energy limits the sample size (Do *et al.*, 2015 ▸; Andrews *et al.*, 2011 ▸) and increases radiation damage (Vojtovà *et al.*, 2019 ▸).

In order to avoid these problems, higher energy is recommended, which is possible using in-line phase tomographic microscopy. As an example, this technique is available at ESRF – the European Synchrotron in Grenoble – on beamlines ID16A (da Silva *et al.*, 2017 ▸) and ID16B (Martínez-Criado *et al.*, 2016 ▸) with energy from 17 keV to 33 keV. Another important issue with nano-characterization is representation. Indeed, reconstructed volumes have to be big enough, and statistical measurement campaigns are often needed due to natural variation in the samples, especially in medical and life sciences. In other words, a large FOV and relatively big samples (a few hundred micrometres) are required as well as fast scans. To increase the scan speed, one option is to increase the X-ray flux, which is a key advantage of synchrotron sources with respect to laboratory ones, but comes at the cost of increased radiation damage. The second option is to use more efficient and faster detectors. There are two kinds of cameras that are widely used for X-ray imaging applications: (i) charge-coupled devices (CCDs) and (ii) complementary metal oxide semiconductor (CMOS) arrays. CMOS technology allows higher frame rates compared with CCD, *i.e.* it speeds up the acquisition for fast scans. Nevertheless, CMOS cameras often present higher noise, which degrades image quality.

Taking all of these parameters (energy, scan time, sample size and FOV) into account, choosing the optimal imaging conditions for 3D nano-imaging becomes a complex task. It is further complicated by the fact that instrument capabilities (achievable resolution and contrast for a given scan duration) are often reported under optimal conditions with high-contrast samples of suitable dimensions. Often only projection images of a bi-dimensional test object with well defined high-contrast features are used, since the manufacturing of a 3D test object with well controlled feature dimensions is not a trivial task in itself. There are, however, many reasons why the cited instrument resolution might not be achievable in practice. Especially in biomedical research, there are often additional constraints, related to the studied system or complementary non-X-ray techniques to be used, which prevent the preparation of samples perfectly dimensioned for nanoCT. On the other hand, scanning samples much larger than the FOV of the instrument (local tomography) can have a further degrading effect on image quality, or require using a higher X-ray energy than what might be expected from simply evaluating the X-ray attenuation and refraction properties of the sample for optimal contrast. It is therefore important to evaluate the impact of different imaging strategies for the practical achievable image quality in a realistic system under study that is subject to the many sample-related non-idealities.

When comparing the quality of two images, the typical metrics used are the spatial resolution, *i.e.* visibility of fine detail in the image, and the signal-to-noise ratio (SNR), or the level of contrast between different materials compared with the magnitude of the noise. The first question to ask when studying image quality in a practical experimental setting is then how do we quantify the resolution and SNR from a 3D dataset depicting a realistic sample system?

In the present paper, we develop a methodology for evaluating the image quality in in-line phase contrast nanoCT scans of a bone tissue sample, using closed porosities within a tricalcium phosphate (TCP) scaffold as a representative system of two known materials. The resolution of the final image is evaluated as the rate of change in the image gray values between the porosities and the scaffold, and SNR is taken as the ratio of the gray value difference between the two materials over the standard deviation within one material. We then use these metrics to explore how different choices made in the experiment design influence the final image quality. By varying the scan time in order to produce projection images of similar intensity in each configuration, we quantitatively compare the effects of the chosen camera technology and the X-ray energy on the resolution and SNR in the reconstructed image.

## 2. Materials and methods

### 2.1. Sample preparation

**Figure 1:**
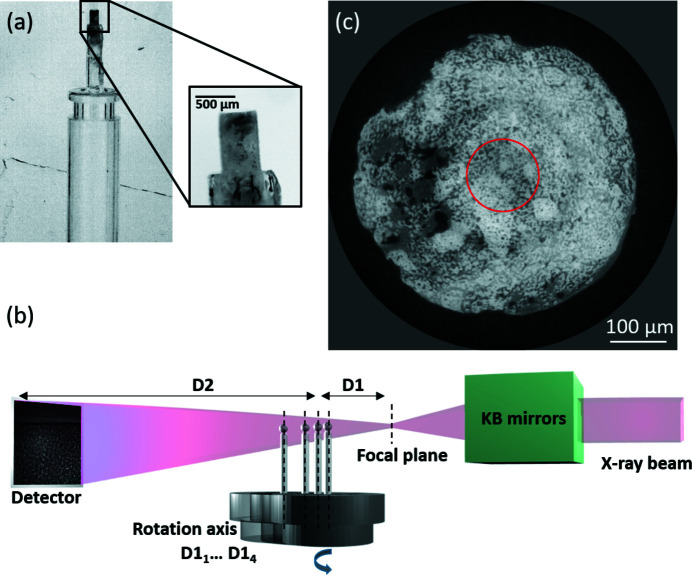
Overall description of the imaging setup and sample. (*a*) Sample mounting. (*b*) Schematic representation of the in-line phase tomographic microscopy setup at the ID16B beamline. (*c*) Cross-section of a reconstruction at a voxel size of 240 nm; the red circle represents the ROI selected for the 50 nm pixel size tomographic scan.

As the basis of the evaluation strategy, we use TCP bone scaffold samples with bone ingrowths (Bohner *et al.*, 2017 ▸; van Lenthe *et al.*, 2007 ▸). We describe here their preparation and implantation, and the preparation of samples for the nanoCT measurements. In brief, the TCP scaffold was implanted into the tibia of a sheep for 6 weeks. More details about the sample can be found in the work by Bohner *et al.* (2005 ▸) – there the sample shown here would be allocated to batch Nos. 4.1 and 4.2. After explant and PMMA-embedding, a sample consisting of a cylinder of 0.5 mm diameter and 1.2 mm length was milled for subsequent nanoCT investigation. For nanoCT scanning, the cylinder was mounted with cyano­acrylate superglue (Loctite precision) on a quartz capillary attached to a brass pin for insertion into the beamline sample stage [Fig. 1 ▸(*a*)].

### 2.2. Synchrotron nanoCT data collection and reconstruction

Synchrotron phase contrast nanoCT was carried out at the ESRF, on the beamline ID16B (Martínez-Criado *et al.*, 2016 ▸). Using the Kirkpatrick–Baez (KB) mirror, the X-rays are focused into a nano beam (50 nm × 50 nm), which then acts as a secondary source producing a conical beam. As described in Fig. 1 ▸(*b*), the camera is at a fixed distance (D1 + D2) from the secondary source. Hence, by adjusting the distance of the sample from this source (D1), the FOV and, as a consequence, the magnification, can be varied. The measurements consist of a quick coarse nanoCT scan with a 240 nm pixel size [Fig. 1 ▸(*c*)] to obtain an overview image of the sample. Then, a region of interest (ROI) was selected vertically at the top edge, and horizontally in the middle of the sample, and scanned with a 50 nm pixel size, which yields a cylindrical 1.4 × 10^−3^ mm^3^ FOV for the PCO edge camera and 0.84 × 10^−3^ mm^3^ FOV for the Frelon camera. This was considered an optimal compromise between resolving the smallest porosities in the TCP scaffold while maintaining a sufficiently large FOV to enable statistically relevant future study to be carried out in a reasonable time frame. Performing high-resolution scans horizontally in the middle of a cylindrical sample is the ideal scenario to alleviate any local tomography related artifacts, even though the projections are highly truncated with the horizontal FOV being 20% or 26% of the sample diameter. In in-line X-ray holotomography (Cloetens *et al.*, 1999 ▸), each nanotomographic scan consists of four CT scans at different propagation distances between the sample and detector for each scan. Four different nanotomographic measurements were performed using two different energies: 17.5 keV or 29.6 keV, and two different detectors: a Frelon CCD camera (Labiche *et al.*, 2007 ▸) or a PCO Edge 5.5 Camera link CMOS (https,//www.pco.de/fileadmin/user_upload/pcoproduct_sheets/pco.edge_55_data_sheet.pdf). For each holotomography scan, the acquisition time per projection was adjusted to keep approximately the same mean intensity (1500–2000 ADU) in the middle of the recorded images for every scan. Acquisition conditions and the camera characteristics are detailed in Tables 2 ▸ and 3 ▸. For each scan 2505 projections were recorded over 360°, with an additional scan of one configuration (CMOS camera at 17.5 keV) performed with 3200 projections.

The data processing to obtain the final 3D volumes used for images analysis is divided into three steps: (i) phase retrieval calculation, (ii) 3D reconstruction and (iii) ring removal. In order to best isolate the effect of the experimental setup on image quality, the phase retrieval and reconstruction workflow was kept identical for all scans. The phase retrieval calculation was completed using an in-house developed octave script (Cloetens *et al.*, 1999 ▸) employing a non-linear conjugate gradient algorithm (Langer *et al.*, 2012 ▸; Weber, 2016 ▸) initialized with a multi-distance (Yu *et al.*, 2018 ▸) Paganin-like approach, and Paganin’s algorithm for the single distance reconstructions (Paganin, 2006 ▸); with a δ/β parameter (ratio of the real and imaginary parts of the complex refractive index, *n* = 1 − δ + *i*β, of the investigated material) of 300 for images acquired at 17.5 keV and 520 for those acquired at 29.6 keV. The tomographic reconstruction was performed using the filtered back projection algorithm with *PyHST2* software developed at the ESRF (Mirone *et al.*, 2014 ▸). In the reconstructed volumes, the remaining ring artifacts were removed using a post-processing *Matlab* (Mathworks Inc. Natick, MA, USA) algorithm based on image filtering (Lyckegaard *et al.*, 2011 ▸).

### 2.3. Image analysis

#### 2.3.1. Quantitative evaluation of image quality

Classic metrics for the quality of a tomographic 3D image are resolution, or visibility of fine details and sharpness of interfaces, and SNR, or the difference in mean gray value between different material phases divided by the amplitude of random noise within a single material. In the case of mineralized tissue on ceramic scaffolds, quantifying these parameters is complicated by two different effects:

(1) Variation in the degree of mineralization of the bone tissue leads to variable gray values within the mineralized tissue. While the mineralized tissue still remains distinct from the scaffold material and unfilled pores in the sample, this variation makes it difficult to calculate the SNR.

(2) A low-frequency variation of the gray values, caused by difficulties in background subtraction due to changing intensity of the X-ray beam during the scan, and by artifacts due to the local tomography approach where, at any given angle, a large proportion of the sample is outside the FOV, affecting the overall intensity of the image but not contributing to the details. This artifact results in a slow variation of up to 20% in gray value (after converting the image to 8-bit format and applying ring artifact removal) over the entire analyzed volume (*e.g.* the gray value associated with the scaffold might be ∼160 in one corner of the analyzed volume and ∼200 near the opposite corner).

To prevent either of these effects from influencing the image quality assessment, we only evaluated the resolution and SNR for isolated pores in the scaffold, *i.e.* pores that are not connected to the main pore space in the sample and thus do not contain any mineralized tissue or PMMA. The two evaluated parameters were then the sharpness of the pore–scaffold interface and the contrast between pore and the surrounding scaffold material.

The *Avizo* software (verson 9.3, Thermo Fischer Scientific, Waltham, MA, USA) was used for semi-automatic segmentation of the dataset and separation of the isolated pores according to the algorithm described in the supporting information. After segmentation, a number of parameters were calculated for each pore identified, including the total number of pixels and sphericity shape factor

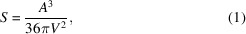

where *A* is the estimated surface area and *V* is the volume of the pore. *S* is independent of the scale of the object, being 1 for a perfect sphere and increasing as the object becomes more irregular. To remove wrongly segmented objects from the analysis, only pores with more than 1000 voxels and *S* < 1.3 were included in the analysis. The resolution and SNR were then determined individually for each remaining pore isolated using the *Matlab* software, based on three datasets:

*D*_1_: labeled data with each isolated pore designated by a unique voxel value.

*D*_2_: binary segmented data, showing the pore space (both isolated and connected pores) and the scaffold material.

*D*_3_: the original volume data (floating-point reconstruction result) without any processing.

In the original volume, each isolated pore appears as a roughly spherical object with higher gray value than the surrounding scaffold. Because of the limited resolution of the image, the partial volume effect and possible blurring introduced by the phase retrieval, the interface is not sharp, but the gray value decreases gradually when moving from the pore to the scaffold. The length of this transition is then a measure of image resolution. In order to compare the resolution between datasets without introducing user error associated with drawing a line in the data, we calculated the mean gray value in the original data (*D*_3_) as a function of distance from the segmented pore surface, in steps of one voxel. This yields a sigmoid-like step-up curve, denoted by *f*. If we model this curve as a convolution between an underlying step function (*i.e.* the true change in density) and a Gaussian smoothing function, this sigmoid curve can be fitted with a suitably parameterized version of the error function,

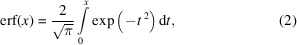

which is a primitive function of the Gaussian.

As the figure-of-merit for the sharpness of the interface (resolution), we adopt the full width at half-maximum (FWHM) of the blurring Gaussian. In the case of equation (2), the FWHM would correspond to 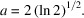 In the actual data, naturally, the difference between the two gray levels is not exactly 1, and the FWHM is different from *a.* Denoting the gray value in the scaffold as *A*_1_ and the gray value in the pore as *A*_2_, the FWHM corresponds to the difference (in distance) of the crossings of the step-up curve with levels

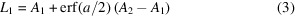

and

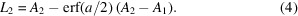

The resolution is then straightforward to determine from the step-up curves as

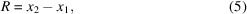

where *f*(*x*_1_) = *L*_1_ and *f*(*x*_2_) = *L*_2_.

For the calculation of SNR, the interface was excluded from the calculation by carrying out a morphological erosion with a spherical structuring element (radius 5 voxels) on both the isolated pore (*D*_1_) and the scaffold material surrounding it (*D*_2_). SNR was then calculated according to the equation

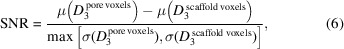

where μ signifies the mean and σ the standard deviation, *i.e.* the SNR is the difference in mean voxel values (in *D*_3_) of the voxels remaining in the pore and scaffold material, divided by the standard deviation of voxel values in either the pore or the scaffold, whichever is higher. Only pores with more than 100 voxels remaining after the erosion were considered for statistical analysis of the results.

This analysis yields quite conservative estimates for SNR and especially for the resolution, but has the advantage of being easily applied to every isolated pore in the dataset without any user input, yielding unbiased comparisons between datasets.

#### 2.3.2. Determination of the bone anatomical parameters

To evaluate and visualize anatomically relevant properties of the sample, a fine segmentation of a smaller sub-region of the best quality scan (Frelon camera, 29.6 keV) was performed using the *Avizo* software, using a watershed-based segmentation complemented with manual editing of the material seeds and the final segmentation result to account for the low-frequency artifact described above. In this segmentation, the mineralized tissue was further separated from the empty pore space, yielding a three-phase segmentation of the sample. Volume, surface area (including and excluding the isolated pores in the scaffold) and specific surface area were then calculated for each component. The detailed segmentation algorithm is presented in the supporting information. *VGStudioMAX* 3.0 software (Volume Graphics GmbH, Heidelberg, Germany) was used for 3D rendering of the results.

## 3. Results

### 3.1. Comparison of image quality parameters between scans

**Figure 2:**
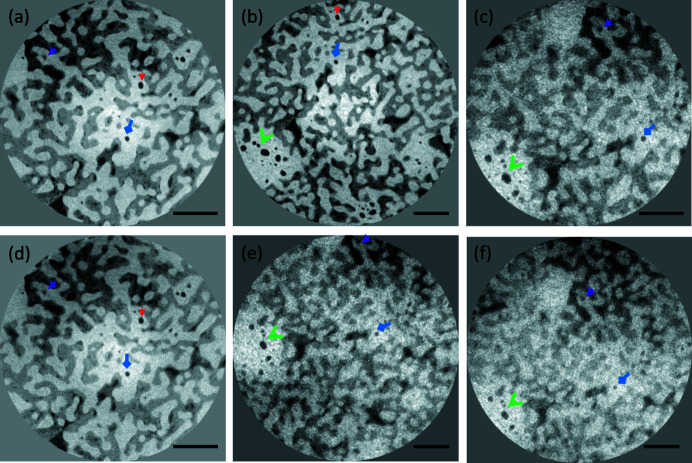
Representative cross-sectional images of the sample under varying image acquisition conditions and phase retrieval methods. (*a*) 29.6 keV, Frelon camera; (*b*) 29.6 keV, PCO Edge camera; (*c*) 17.5 keV, Frelon camera; (*d*) 29.6 keV, Frelon camera, phase retrieval from a single distance; (*e*) 17.5 keV, PCO Edge camera, 3200 projections; (*f*) 17.5 keV, PCO Edge camera, 2505 projections. The different arrows highlight the common features from one reconstruction to another.

The reconstructed data of all configurations enabled us to see the three phases of our investigated sample, namely newly formed tissue, scaffold and air-filled pores (Fig. 2 ▸).

However, the image quality difference between the various reconstructions is striking even to the naked eye. In particular, the reconstructions of data acquired with the lower X-ray energy (17.5 keV) appear noisy, with contours of especially the bone/mineralized tissue phase difficult to discern. The measured SNR and resolution, as defined in the previous section, ranged from 8.4 ± 1.4 nm and 400 ± 20 nm to 4.4 ± 0.5 nm and 540 nm ± 60 nm in the highest-quality reconstructions. Note that the resolution 540 ± 60 nm corresponds to the low-energy scan with the PCO camera, but with an increased number of projections (3500) compared with the other scans. With 2500 projections, the image acquired in these conditions is too noisy to accurately segment the shape of many closed pores; the estimations of SNR and resolution reported in Table 4 ▸ for this scan are based on just 15 individual pores, and should only be considered indicative of the actual scan quality. The measured SNR for the 3200 projection (4.5 ± 0.7) scan is within the margin of error from the lowest measured value (Frelon camera at 17.5 keV). For the same energy and average image intensity, the image quality obtained with the Frelon camera is slightly superior to the PCO camera. Although the PCO Edge camera yields a slightly better SNR value with 29.6 keV X-ray energy, the Frelon has a more noticeable advantage in terms of resolution. On the other hand, the impact of the energy of the incident beam on image quality is more significant; for both cameras, the images obtained at 29.6 keV are better quality than those obtained at 17.5 keV (Table 4 ▸).

**Figure 3:**
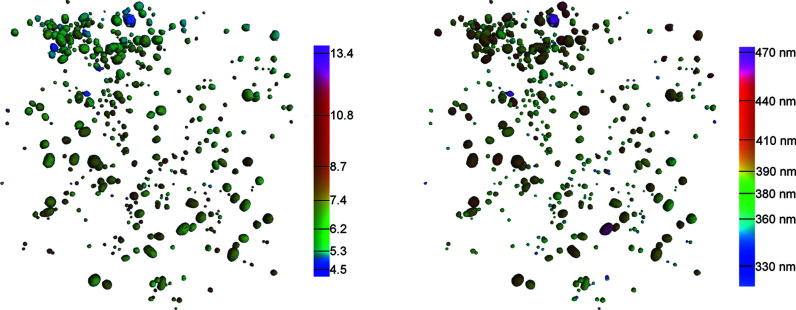
3D renderings of all analyzed isolated pores in scan Bone3 (Frelon camera, 29.6 keV). Colors correspond to the measured SNR (left) and resolution (right). The size of the visualized volume is 68 µm × 71 µm × 65 µm.

On ID16B, the alignment of the beamline when changing the X-ray energy or camera configuration requires using the sample stage for reference objects. It was therefore not possible to maintain the sample alignment between the scans, and with the local tomography approach used here it was not feasible to image exactly the same section of the sample with each investigated setup. This raises the question of the influence of the pore size on the SNR and resolution measured with the above methods. Statistical analysis of the 359 pores detected in the best-quality scan (Frelon camera, 29.6 keV), however, does not reveal any strong correlations between pore size and resolution or SNR. This can be visually confirmed in Fig. 3 ▸, which shows a 3D rendering of the pore space, with the pores colored according to their SNR or resolution values.

### 3.2. Analysis of specific surface area

**Figure 4:**
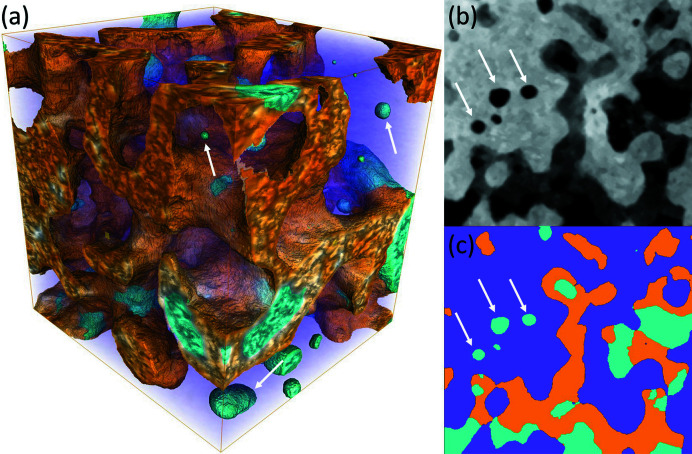
Analysis of anatomical parameters from the data. (*a*) 3D rendering of the sample structure from scan Bone3 (Frelon camera, 29.6 keV X-ray energy) after segmentation. The scaffold material is shown in a transparent purple hue, the bone in orange and porosities in cyan. Size of the visualized volume is 22 µm × 20 µm × 22 µm. (*b*) Example gray value slice through this cropped dataset. (*c*) Segmentation corresponding to image (*b*), with the same coloring as in (*a*). White arrows highlight isolated porosities considered for image quality analysis, *i.e.* roughly spherical objects of the porosity phase that are not in contact with the bone.

To demonstrate the biologically relevant information that can be extracted from nanoCT data, a detailed analysis of the bone anatomy was carried out on the highest quality scan. According to the above image quality analysis, the scan using the Frelon camera and 29.6 keV X-ray energy was chosen for this purpose. Fig. 4 ▸ illustrates the microstructure of the sample: the scaffold material is rendered in a transparent purple hue to reveal the newly mineralized bone tissue rendered in orange and the void space rendered in cyan. From an application point of view, the main interest in the analysis of the nanoCT investigation is to confirm the ingrowth of mineralized tissue (MT) into the porous TCP. In addition, we report the specific surface area of the scaffold material, which is an important consideration for the growth rate of bone tissue. Here, the specific surface area is defined as the surface area within a unit bulk volume. However, as Fig. 4 ▸ illustrates, the scaffold also contains isolated closed voids that are disconnected from the main porosity, and therefore inaccessible for bone growth. Counting the entire surface area of the scaffold, the specific surface area for the scaffold phase is 0.47 µm^−1^; excluding the closed porosities from the analysis, this value drops to 0.45 µm^−1^, which means the closed porosities have a small but measurable effect on this parameter. The mineralized tissue covers 83% of the available scaffold surface. The surface to volume ratio of the scaffold phase was found to be 0.780 µm^−1^ or 0.760 µm^−1^, depending on whether the closed porosities were included in the surface determination or not.

## 4. Discussion

The most important point to note of the measured image quality parameters is that, by keeping the overall image gray value constant, it does not make a great difference whether a CCD or CMOS camera is used for holotomography acquisition. While the reconstructions obtained with the Frelon camera are arguably of slightly better quality (comparable SNR with somewhat improved resolution), this advantage could easily be overcome by the PCO Edge camera by simply increasing the acquisition time per projection or the number of projections. In the chosen imaging conditions, we estimated that the PCO Edge used ∼10% of the dose of the Frelon camera to form the image. Additionally, imaging at 29.6 keV resulted in a relative dose of ∼30% compared with imaging at 17.5 keV. Thus, imaging with the PCO Edge at 29.6 keV required 3% of the dose required with the Frelon camera at 17.5 keV. However, no dose-related effects were observed during the experiments. It is notable from the scans with the PCO Edge at 17.5 keV that, by increasing the number of projections by 28%, the measured SNR is improved by 10% and the measured resolution by 8%. The scans reported here using the PCO Edge camera were faster by a factor of 2.8–7.2, which means that there is room for significant improvement while still saving in terms of both scan time and sample dose compared with the Frelon camera.

The chosen X-ray energy had a much bigger influence on the image quality than the camera choice. For both cameras, scans at the lower energy have 46% worse SNR and 23% worse resolution compared with the higher energy scans. This effect is more related to the sample size and consequent attenuation than the X-ray properties of the sample material. The main physical quantity measured in holotomography is δ, the deviation from unity of the real part of the complex refractive index (*n*).

In the specific use case discussed here, the X-ray properties of the sample are rather homogeneous. Bone is a highly hierarchical material with different mechanical properties and composition for the different length scales (Fratzl & Weinkamer, 2007 ▸). The volume fraction of the main mineral component, hy­droxy­apatite (HA), is less than 55% and depends on the level of mineral maturation (Fratzl *et al.*, 2004 ▸).

The δ values for HA and the TCP scaffold are very similar at both X-ray energies used: theoretical values of *δ*_HA_ are 2.15 × 10^−6^ at 17.5 keV and 7.47 × 10^−7^ at 29.6 keV, compared with *δ*_TCP_ = 2.13 × 10^−6^ and *δ*_TCP_ = 7.41 × 10^−7^, respectively, for the scaffold phase. The main contrast observed between the mineralized tissue and the scaffold is therefore due to incomplete mineralization and the presence of other components such as collagen in the newly mineralized bone tissue.

In air, both δ and β (the imaginary part of *n*) are negligible compared with the scaffold values, and one could expect the higher δ values at 17.5 keV to improve the SNR measured with the above-described methodology. This is, however, not the case here, which is likely due to the effects of strong attenuation induced by the sample. Average X-ray transmission through the sample at the lower energy is only 45% (measured as mean *I*/*I*_0_ in one projection image, *cf*. Table 4 ▸) and less than 5% at the minimum. In the higher energy scan, average transmission is 83% (9% minimum). In standard, attenuation based tomography (thus measuring β instead of δ), the 45% transmission would indeed be closer to the commonly used ideal value of approximately 14% [see *e.g.* Reiter *et al.* (2012 ▸) for an empirical discussion], and expected to yield images with improved contrast to noise ratio. In in-line phase-contrast imaging, however, the X-ray attenuation is not in itself a quantity of interest, but rather represents a part of the signal that needs to be decoupled from the phase information in the phase retrieval step of the analysis. On the other hand, phase contrast manifests itself in the projection images as dark and light fringes around interfaces, and the phase retrieval step of holotomography essentially means transforming the magnitude of these fringes into numerical values for the phase change of the X-rays traversing the sample. Two phenomena could contribute to the reduced image quality at the lower energy. At strong absorption and low Fresnel numbers, the non-linear contribution to the contrast is greater (Cloetens *et al.*, 1999 ▸). This makes phase retrieval with linear approaches more difficult. Despite refinement with a non-linear algorithm, the initialization provided by the linear phase retrieval could be too far from the desired solution, so that the non-linear conjugate gradient algorithm finds a local minimum. The other contribution could be due to the combined effect of strong attenuation and strong phase contrast bringing the intensity in the dark fringes close to zero, thus possibly increasing the effect of noise and detector non-linearities on the phase retrieval.

From a bio-material point of view, the nanoCT analysis of the sample presented could help to demonstrate that bone can actually grow into pores as small as a few micrometres (Bohner *et al.*, 2017 ▸). Though 3D assessment of bone ingrowth into scaffold materials or bone and scaffold interaction have been widely addressed on the micrometre scale or above (Jakus *et al.*, 2016 ▸; Jones *et al.*, 2007 ▸, 2009 ▸; Peyrin, 2011 ▸), most previous studies on the sub-micrometre scale rely on 2D imaging modalities (Bernstein *et al.*, 2013 ▸; Lewin *et al.*, 2017 ▸; Palmquist *et al.*, 2012 ▸; Shah *et al.*, 2019 ▸). Synchrotron nanoCT is thus a complementary modality for the analysis of the interaction of scaffold and bone tissue. Beyond bone growth into scaffold porosities, we here demonstrate the assessment of the bio-material surfaces available for the ingrowth of bone tissue and quantify the specific surface area. We speculate that material architecture is not only relevant in terms of mechanical performance but also on how newly formed tissue can integrate into the bio-material. Thus the (open) pore distribution defines the permeability for newly formed bone tissue penetration. For bio-degradable materials, such as the biomaterial used within this study, the available surface can interact with newly formed bone and this available biomaterial surface is the most potent site for material exchange. In human bone, the surface of vessel pores per bone volume is about 0.002–0.005 µm^−1^ (Hesse *et al.*, 2014 ▸) while the surface density of the sub-micrometre cellular network (LCN) is on the order of magnitude of 0.1 µm^−1^ (Hesse *et al.*, 2015 ▸). The surface density found for the TCP sample analyzed in this study reveals a surface density of 0.76 µm^−1^. Tuning the pore size distribution and the specific surface area of degradable bone grafts should influence the penetration of newly formed bone tissue and degradation rate. Theoretical prediction of surface density of bone substitutes on the sub-micrometre scale is challenging and therefore the experimental characterization of the 3D architecture of bone substitutes, including the surface density of the sub-micrometre level, may be of high relevance to better understand the impact of material architecture and material integration into the bone on the macro-scale.

## 5. Conclusions

We have presented a framework for evaluating the impact of the imaging system on the final image quality in imaging of mineralized artificial bone grafts. Evaluating image quality in this type of sample is made more difficult by the complex volumetric structure and the spatial distribution of two different phases. We proposed a procedure to extract quality measures such as the SNR and spatial resolution. The procedure was applied to imaging at the ID16B beamline at the ESRF. This allowed us to choose appropriate imaging parameters for a detailed analysis of the sample in terms of pore volume, bone ingrowth ratio and volume to surface ratios of the different phases. Thus, the proposed evaluation strategy can be used to optimize the imaging parameters in the imaging of bone scaffold samples. The procedure can be extended to other similar imaging problems, where reasonably smooth and homogeneous objects of one material are embedded in another material, such as precipitates in a metal matrix or pores in solid oxide fuel cells.

## 6. Related literature

The following reference, not cited in the main body of the paper, has been cited in the supporting information: Buades *et al.* (2005 ▸).

## Supplementary Material

Details of the data analysis with the Avizo software. DOI: 10.1107/S1600577522003137/gy5031sup1.pdf

## Figures and Tables

**Table 1:** Examples of nano-resolution beamlines offering X-ray nano-imaging around the world Minimum pixel size and/or achievable spatial resolution are given. FZP: Fresnel zone plate.

Synchrotron	Beamline	Pixel size (nm)	Resolution (nm)	Energy (keV)	Setup	Reference
Diamond Light Source	I13L	25	50–100	6–13	FZP	Rau *et al.* (2019 ▸)
Petra III (DESY)	P05	–	50	9–14	FZP	Greving (2019 ▸)
Swiss Light Source	TOMCAT	60	150–200	8–20	FZP	Stampanoni (2021 ▸)
Synchrotron SOLEIL	Anatomix	20	80	10	FZP	Weitkamp *et al.* (2017 ▸), Weitkamp (2021 ▸)
Advanced Light Source (ALS)	6.1.2	–	15	0.3–1	FZP	Mi-Young (2021 ▸)
Stanford Synchrotron Radiation Laboratory (SSRL)	BL6-2c	–	30	2.3–17.5	FZP	Andrews *et al.* (2008 ▸), Liu (2021 ▸)
Advanced Photon Source (APS)	32-ID-C	–	10	8–9	FZP	De Andrade *et al.* (2021 ▸)
National Synchrotron Light Source II (NSLS II)	18-ID	–	30	6–10	FZP	Ge *et al.* (2018 ▸), Lee (2021 ▸)
SPring-8	BL47xu	38	200	6–12	FZP	Furuta *et al.* (2018 ▸), Takeuchi *et al.* (2011 ▸)
MAX IV	NanoMax	6–45	79–155	6–22	KB mirrors	Kalbfleisch *et al.* (2022 ▸)

**Table 2:** Acquisition parameters

Scan	Energy (keV)	Camera model	Camera exposure time per projection (s)	Total scan time
Bone3	29.6	Frelon	1.8	6 h 7 min
Bone5	29.6	PCO edge	0.25	51 min
Bone7	17.5	Frelon	0.18	1 h 24 min
Bone8	17.5	PCO edge	0.05	26 min
Bone6†	17.5	PCO edge	0.05	30 min

† 3200 projections.

**Table 3:** Camera characteristics

	Frelon	PCO edge 5.5
	CCD	CMOS
Array size	2048 × 2048	2560 × 2160
Pixel size	24 µm	6.5 µm
Dynamic range	16000	27000
Electronic noise sensitivity	33 e^−^ r.m.s., 9.3 e^−^/adu	1.5 median/1.7 r.m.s. e^−^
Integral non-linearity	±0.3% of full range	<1%
Full well capacity	275000 e^−^	30000 e^−^
Quantum efficiency	24%	60%
Dark current	1 e^−^ pixel^−1^ s^−1^	2 e^−^ pixel^−1^ s^−1^
Resolution	16 bit	16 bit
Read out speed	40 Mpixel s^−1^	572.0 Mpixel s^−1^
Scintillator used	LSO:Tb, 20 µm-thick	LSO:Tb, 17 µm-thick

**Table 4:** Comparison of SNR, resolution and transmission for the different conditions of acquisitions

Camera	Energy (keV)		SNR mean ± STD	Resolution mean ± STD	Number of porosities	Average transmission in projection ± STD (every 100th projection)	Pixel-wise transmission in single projection ± STD (first projection of scan)
PCO	29.6	Four distances	8.4 ± 1.4	440 ± 30	846	0.83 ± 0.11	0.83 ± 0.51
	17.5	Four distances (3200 projections)	4.5 ± 0.7	540 ± 60	46	0.45 ± 0.09	0.44 ± 0.41
	17.5	Four distances (2505 projections)	4.1 ± 0.5	590 ± 70	15	–	–
Frelon	29.6	Four distances	8.2 ± 1.7	400 ± 20	359	0.83 ± 0.11	0.84 ± 0.49
	17.5	Four distances	4.4 ± 0.5	490 ± 50	113	0.46 ± 0.1	0.47 ± 0.41
	29.6	One distance	5.2 ± 1.4	460 ± 50	359	–	–
