# Supplementary material for: Evaluation of imaging setups for quantitative phase contrast nanoCT of mineralized biomaterials
Source: J Synchrotron Radiat. 2022 Apr 25;29(Pt 3):843–52. doi: 10.1107/S1600577522003137 (PMC9070718; doi:10.1107/S1600577522003137)
Supplement: Supplementary file 1 [file s-29-00843-sup1.pdf]

JOURNAL OF SYNCHROTRON RADIATION

Volume 29 (2022)

Supporting information for article:

Evaluation of imaging setups for quantitative phase contrast nanoCT of mineralized biomaterials

Jussi-Petteri Suuronen, Bernhard Hesse, Max Langer, Marc Bohner and Julie Villanova

Details on the data analysis with Avizo software

After conversion of data to 8 bit volumes and ring artefact removal, segmentation of isolated pores in the sample volume implemented the following algorithm:

1. Mask the dataset to remove non-reconstructed areas outside the (cylindrical) field-of-view.
2. Filter data with the Non-local means filter (Buades et al. 2005) applied to the XY planes, with a search window of 21 voxels, local neighborhood of 9 voxels and similarity value of 0.8
    a. In the case of noisier datasets acquired with 17.5 keV X-ray energy, also perform a bilateral filtering with a 9 x 9 x 9 voxel kernel and similarity value of 40.
3. Perform a bottom-hat transform (i.e. subtraction of the original image from its morphological closing) with a spherical structuring element of radius 100 voxels (i.e. 5 µm)
4. Perform a dual (hysteresis) thresholding on the bottom-hat result, setting the lower threshold after the main peak in the histogram of the bottom-hat data (corresponding to the scaffold), and the second threshold just above the top of the next peak (corresponding mostly to mineralized tissue surrounded by scaffold, and/or empty pore volume surrounded by mineralized tissue): include in the pore space all voxels with value above the higher threshold, and voxels with values between the two thresholds if they are connected to a retained voxel by at most 50 voxels.
5. Check the segmentation result for obvious large errors, and correct them manually. The result of this step is dataset D_2 discussed in the image processing section of the article.
6. Apply a border kill operation on the segmentation result, removing all pores that touch the edges of the dataset. This removes the main accessible pore volume (including all mineralized tissue), and all the isolated pores that are only partially included in the analyzed volume.
7. Apply a labeling to the remaining pores, giving each a unique identifier. The result of this step is dataset D_3 discussed in the image processing section of the article.
8. For each labeled pore, calculate the volume, surface area, bounding box, mean value, and shape factor of the pore. These values are used to exclude too small or abnormally shaped (either as a result of segmentation error or fusing several smaller pores) pores from the analysis.

For detailed analysis of anatomical parameters, a more detailed segmentation was necessary to also separate the mineralized tissue from the empty pore volume. As there are now three different phases to segment, simple thresholding of bottom-hat or top-hat transforms is not sufficient to distinguish the phase with middle gray value (mineralized tissue) from the two others. Moreover, due to low-frequency artefact, the gray value ranges of the different phases overlap, necessitating a more

complex segmentation approach. The idea is to do a seeded watershed segmentation, finding voxels that are darker than their surroundings as seeds for the pore phase, brighter voxels for the scaffold phase, and intermediate value voxels for the mineralized tissue phase:

1.-2. As above, cropping the dataset more tightly to reduce the effect of the low-frequency artefact.

3. Calculate the 3D gradient amplitude of the filtered data using the Canny-Deriche edge detector.

4. Perform a closing on the edge detection result using a spherical structuring element with radius 5 voxels.

5. Choose only voxels with value less than 3 in the closed edges to designate 'flat' areas in the volume.

6. Perform a bottom-hat transform and hysteresis thresholding as 3.-4. above, but in order to identify only certain pore voxels (excluding mineralized tissue), set the thresholds before the highest peak in the histogram, corresponding to the lowest regions in the image. Also set the connectivity criteria to 5 voxels instead of 50. The result will be the seed voxels for the 'pore' phase in the segmentation.

7. Repeat phase 6., but do a top-hat transform (i.e. subtraction of the opening of the image from the original) instead of bottom-hat to get seed voxels belonging to the scaffold.

8. Choose a narrow range of intermediate-value voxels as belonging to the mineralized tissue by simple thresholding.

9. Combine all 3 seed voxel datasets into one, edit manually to remove gross errors and add voxels to the seeds where necessary.

10. Restrict the seed voxel dataset to the flat areas calculated in step 5.

11. Perform a watershed segmentation with the seed dataset as input, and the closed gradient image (step 4) as the elevation image.

12. Edit the watershed result manually to remove major errors, and assign the watershed lines to different phases by successive dilation of all 3 phases into the set of unassigned voxels.

13. For each phase, calculate total volume and surface area.

References

Buades, A., Coll, B. and Morel, J.M, (2005), *A non local algorithm for image denoising*, in *Proc. Int. Conf. Computer Vision and Pattern Recognition (CVPR)*, **2**, 60-65.
